# Limbic Encephalitis as a Late Complication of Relapsing Polychondritis: A Case Report and Review of the Literature

**DOI:** 10.31138/mjr.34.2.229

**Published:** 2023-06-30

**Authors:** Vasiliki Michalaki, Dimitrios Katsifis-Nezis, Tzim Rallis, Konstantinos Kanavouras, Zisis Tsouris, Andromachi Roussopoulou, Maria Ismini Arvaniti, Anastasia Skafida, Smaragdi Katsoulakou

**Affiliations:** Peripheral General Hospital of Peiraias “Tzaneio”, Peiraias, Attiki, Athens

**Keywords:** limbic encephalitis, relapsing polychondritis, rapidly progressive dementia, vasculitis, cyclophosphamide

## INTRODUCTION

Relapsing polychondritis (RP) is a rare connective tissue disease characterized by recurrent inflammation of cartilaginous and proteoglycan-rich tissues. Auricular and nasal chondritis, as well as oligo- or polyarthritis are the clinical hallmarks of the disease, presenting in over 80% of cases. However, multiple organs can be affected, such as the eyes, the cardiovascular system, the skin and the nervous system, highlighting the systemic nature of the disease. In the majority of patients RP shows an abrupt onset with a progressive course and frequent relapses, resulting in destruction and functional impairment of the affected structures. Its incidence is estimated between 0.71 and 3.5 cases per million, while both genders are equally affected.^1^ The exact pathogenesis of the disease remains unknown. Current understanding implicates both cell mediated and humoral immune systems. HLADR4 is identified as a key allele, though no ethnic or familial clustering have been noted. To date, diagnosis relies on diagnostic criteria proposed by McAdam and colleagues in 1976 and their modification by Damiani et al. and Michet et al.^2,3,4^ Laboratory, histological, or imaging methods are supportive but lack diagnostic specificity.^5^

Central nervous system (CNS) involvement in RP is a life-threatening, but infrequent manifestation of the disease accounting for 3% of the reported cases.^6^ Neurologic involvement manifests with heterogenous features including limbic encephalitis, aseptic meningoencephalitis, dementia, and cranial nerve palsies. We report a case of a patient with known RP of distant onset that presented with rapidly progressive dementia (RPD) secondary to limbic encephalitis and concomitant parenchymal vasculitis. In our knowledge this is the first reported case of CNS involvement in RP with presentation ten years after disease onset, exemplifying the diversity of this condition in terms of clinical appearance and time course.

## CASE DESCRIPTION

A right-handed, 71-year-old Caucasian male was admitted to our hospital for gradual cognitive decline associated with personality changes that had appeared over the previous 6 months. The patient’s sister described signs of disinhibition and emotional instability, unsteady gait, disorientation, and progressively deteriorating memory problems. His past medical history revealed smoking (40 pack years), hypertension, and dyslipidaemia treated with amlodipine/valsartan and rosuvastatin respectively. Ten years earlier, he had demonstrated painful auricular erythema and swelling, as well as ocular inflammation and was eventually diagnosed with RP based on clinical features and auricular biopsy findings. He was treated with azathioprine, which he discontinued on his own 6 months prior to his visit. His family history was significant regarding an unspecified, late-onset dementia of his mother. His three siblings had a negative history of neurologic or autoimmune disorders, except of his sister who suffered from rheumatoid arthritis.

Physical examination on admission was unremarkable, with no evidence of arthritis, skin exanthem or ocular irritation. Signs of active auricular or nasal inflammation were absent. However, the pinna on both sides seemed flattened and atrophic, whereas his voice was hoarse. Overt focal deficits were not observed. On standing and walking, a wide-based gait with a positive Romberg test were noted. During the examination the patient remained alert, however he showed a euphoric disposition, often displaying agitation and expressing inappropriate remarks. Neuropsychological testing revealed pronounced disturbances in short-term memory, as well as recall, orientation, and executive functions.

**Figure 1A-1B-1C.**
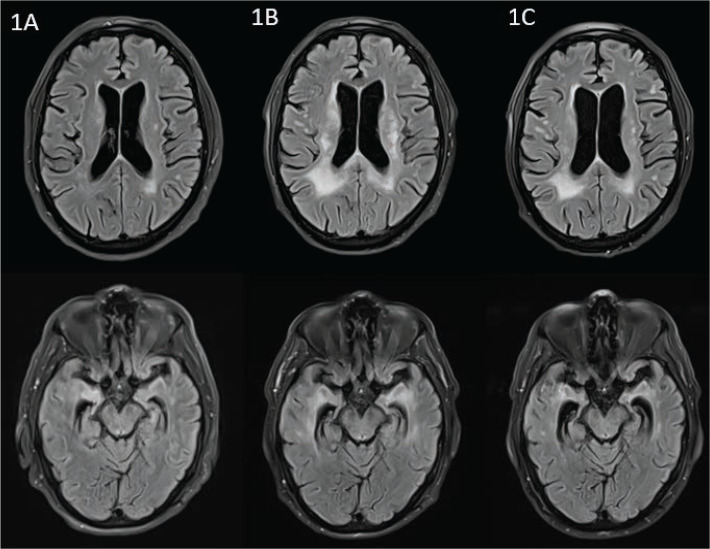
**Α)** MRI findings on presentation: T2-weighted and fluid-attenuated inversion recovery (FLAIR) images reveal hyperintensity in the medial temporal lobes, non-specific hyperintense small-diameter lesions in sub-cortical and deep white matter, **Β)** Cerebral MRI one month after initiation of corticosteroid therapy showing expansion of hyperintense areas in the medial temporal lobes with accompanying atrophy, increased lesion load in periventricular and deep white matter, **C)** Cerebral MRI two months later after initiating cyclophosphamide showing decreased lesion load in periventricular and deep white matter, attenuated hyperintensity in temporal lobes accompanied by significant atrophy.

Brain MRI showed diffuse small-diameter focal lesions in T2 and FLAIR sequences located in the sub-cortical and deep white matter, as well as hyperintensity in both medial temporal lobes (**Figure 1A**). Diffusion weighted imaging was negative for recent infarction. Cerebral MR angiography with complementary high-resolution vessel wall imaging excluded the presence of aneurysms or arterial stenoses in the circle of Willis. Vessel wall enhancement was not noted.

Initial laboratory investigations were unremarkable except for a mild elevation of the erythrocyte sedimentation rate (40 mm/hr). A lumbar puncture was performed, and cerebrospinal fluid (CSF) examination demonstrated a lymphocytic pleocytosis (118/mm^3^, lymphocytes=83%) with increased total protein (100mg/dl) and marginally reduced glucose concentration (53 mg/dl, serum glucose: 117 mg/dl). A comprehensive investigation for infectious agents was negative, including herpes simplex virus I and II, CMV, Epstein-Bar virus, Varicellazoster virus, Human herpesviruses 6, 7 and 8, Listeria Monocytogenes, Cryptococcus Neoformans, JC virus, Borrelia burgdorferi/miyamotoi and Mycobacterium Tuberculosis. CSF cytology did not detect malignant cells. Notably, Quantitative immunoglobulin analysis revealed intrathecal IgG synthesis (IgG index =1.14, presence of oligoclonal bands).

Serum screening for HIV, syphilis, or hepatitis was negative. Further investigations, such as serum angiotensin-converting enzyme, Mantoux reaction, and interferon-γ assay, testing for autoimmune diseases (ANA, ANCA, C3/C4, RF, Ig4, ENA), antibodies for paraneoplastic (Anti-Amphiphysin, Anti-CV2, Anti-Hu, Anti-PNMA2, Anti-Recoverin, Anti-Ri, Anti-SOX1, Anti-Yo, Anti-zic4, AntiTr) and autoimmune encephalitides (NMDAR, GAD, ΑMPAR, GABAR, DPPX, GluR5, Anti-CASPR2, Anti-LGI1, Anti-VGKC) were also without positive results. Finally, an extensive screening for systemic malignancy, including gastroscopy/colonoscopy and computed tomography of the chest and abdomen was unfruitful.

Based on the clinical features, the MRI and CSF findings, as well as on the exclusion of pertinent alternative diagnoses, we concluded that limbic encephalitis secondary to relapsing polychondritis was an important consideration.

Therapy was promptly initiated with seven daily pulses of intravenous methylprednisolone (MP) (1gr/day), followed by oral prednisone (60mg/d). On follow-up one month later, our patient showed substantial clinical response, with improved orientation and attention on testing with cognitive batteries and a significant attenuation of CSF pleocytosis (18/mm^3^) with still increased total protein (80mg/dl) and normal glucose concentration (86mg/dl, serum glucose 113mg/dl). Surprisingly, a new brain MRI displayed further enlargement of the hyperintense areas in the medial temporal lobes, with signs of atrophy. Additionally, lesion bulk in the white matter and basal ganglia was increased, strongly indicating a vasculitic process (**Figure 1B**).

Deterioration on imaging prompted the addition of a second-line immunomodulatory agent. Cyclophosphamide was initiated as induction therapy (750mg/m2 monthly for 6 months) and after completion, it was substituted with azathioprine as maintenance treatment (3mg/kg/d). A follow-up brain MRI two months into treatment with cyclophosphamide demonstrated a significant reduction of vasculitic lesions (**Figure 1C**). However, the progression of atrophy in the hippocampi and temporal horns was not halted, possibly reflecting the inevitable end-product of a long-lived inflammatory process.

Currently, three years after initial presentation, the patient is clinically stable, still displaying anterograde amnesia. However, he is able to carry out rudimentary daily activities with minimal supervision. He remains on azathioprine therapy and prednisone was slowly tapered to 5mg/day.

## DISCUSSION

**Figure 2.**
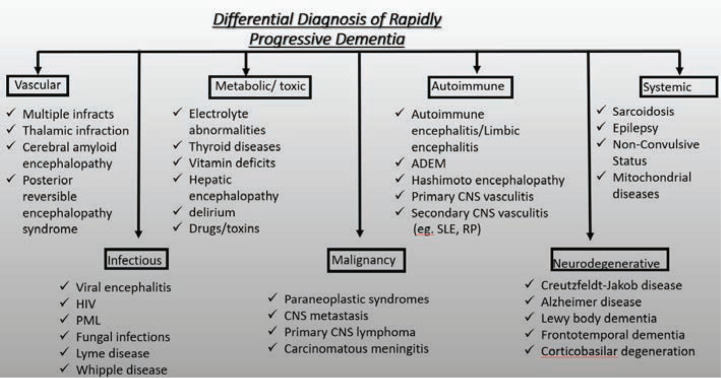
Early differential diagnosis of rapidly progressive dementia in our patient^12,13,14^

Relapsing polychondritis is an uncommon disease first described in 1923 by Jaksch-Wartenhorst. The term was coined by Pearson and colleagues in 1960, in order to describe the undulating course of this condition.^7,8^ CNS involvement is rare, affecting approximately 3% of patients. It can appear as part of the initial clinical syndrome,^9^ occasionally as the presenting symptom, 10,11 or, in the case of our patient, as a late complication of the disease. Due to the long period separating the time of initial RP diagnosis and the onset of cognitive symptoms (10 years), as well as the lack of apparent disease activity, a direct association could not be drawn. Initially, our differential diagnosis included vascular, metabolic, autoimmune, systemic, infectious, malignant, and degenerative causes (**Figure 2**).

Due to the rarity of the syndrome most data are derived from case reports, while only a few case series exist.^15,16^ Currently, there are no diagnostic criteria of CNS involvement in RP; hence, it is a diagnosis by exclusion. The pathophysiology underlying CNS involvement remains unknown and prevailing theories favour a vasculitic process.^17,18^ In 2017, Ellis et al. reported two RP cases complicated by cognitive decline that reflected two separate etiopathogeneses. The first case described a patient with fulminant cognitive dysfunction displaying vasculitic parenchymal foci on MRI and histopathologic evidence of small-vessel infiltration from atypical multi-nucleate giant cells. In contrast, the second case noted a more insidious course with behavioural changes and memory deficits resembling the clinical complex of limbic encephalitis. Brain MRI in this patient showed atrophy of the medial temporal lobes.^19^ Interestingly, our patient, presented with a combination of these two phenotypes, expressing both the neuropsychiatric features and medial temporal lobe pathology of limbic encephalitis, together with wide-spread vasculitic lesions on MRI.

We searched PubMed, Web of Science and Google Scholar in December 2021 using “relapsing polychondritis” AND (“limbic encephalitis” OR “encephalitis” AND “limbic system”) AND “case reports” as the keywords. We retrieved a total of 20 articles, where 13 met the inclusion criteria (English, limbic encephalitis) (**Table 1**). The titles and abstracts were screened by 2 authors and those that did not meet the inclusion criteria were discarded. Full texts of the remaining citations were obtained and examined for eligibility. Reference lists of retrieved articles were manually screened for potentially relevant case reports.

**Table 1.** Articles retrieved within inclusion criteria.

**Ref.**	**Author/Year**	**Age/gender**	**symptoms**	**lab**	**MRI**	**EEG**	**CSF**	**Abs**	**Biopsy**	**treatment**	**Outcome**	**relapse**
17	Ohta Y et al./2004	57/M	1^st^ admission: Bilateral hand arthritis, nystagmus HL 2^nd^admission: FV, vertigo, HA, Kerning sign, ASAT, CJ immediate MI	↑	1^st^: Normal 2^nd^: Τ2/FLAIR hyperintensity in bilateral HC/AB/PVWM while Gd-DTPA enhanced lesions in PVWM 3^rd^: new hyperintensity in bilateral BG	diffuse slow waves (5–6 Hz) with slow α-activities (8–9 Hz)	67 cells 80% lymph, Glu normal IgG index 1.43.	No	No	IVMP, PPO	IMP	No
15	Fujiki F. et al./2004	45/M	CJ, FV, AG, weight loss, C, euphoria, DO, MI, SA, hyperactivity, ASAT	↑	1^st^: Τ2 hyperintensity in bilateral medial TL, HCs, and insular cortex 2^nd^: (1 month) bilateral cortical atrophy in the TL with dilated temporal horns	N/Α	8 cells 94% lymph, Glu normal IgG index 1.02.	N/Α	Yes	IVMP, PPO	IMP	No
15	Fujiki F. et al./2004	62/M	MI, C, AG, euphoria, ASTAT, HL, confabulation	↑	1^st^: Τ2/FLAIR hyperintensity in bilateral TL/insula/HC/amygdala/DWM 2^nd^: (2 months) bilateral cortical atrophy within the frontotemporal lobes	N/Α	24 cells 83% lymph, Glu normal IgG index 1.56	N/Α	Yes	IVMP, PPO	Mild IMP	No
18	Yan M et al./2007	53/M	MI, aggression, problem solving difficulties, ASAT, SA		1^st^: N 2^nd^: (5months) MRI Hyperintensity in L TL involving the uncus and HC	1^st^: N 2^nd^: periodic sharp wave complexes every 1.0–1.5 seconds	N/a	N/a	Yes	Unspecified steroids	death	N/a
20	Kashihara K et al/2009	62/M	Seizures, ASAT, FV, delirium, A, clonic convulsion in the left face and upper and lower limbs, HL		1^st^: FLAIR MRI slightly hyperintensity in medial TL/HC/amygdala and mild chronic ischemic change in the putamen 2^nd^: (14 months) medial TL atrophy	Background activities to 4–5 Hz.	512 cells 30% lymph, Glu N/a IgG level of 8.7 mg/L	GluR N2B (CSF and serum)	No	IVMP, PPO	Slight IMP	Yes
21	Storey K et al./2011	73/M	C, FV, ASAT, CI, A Confabulation, gait instability, myoclonic jerks, hallucinations, seizures		FLAIR/T2 hyperintensity in TL/HC, vascular white matter lesions and moderate atrophy	slow waves in the temporal regions.	89 cells mono, Glu N/a	No	No	IVMP	death	N/a
27	Wang JC et al./2011	44/M	MI, irritation	↑	1^st^: T2 hyperintensities in L HC 2^nd^: R TL and R PVWM	N/a	Mononuclear pleocytosis, Glu N/a	No	No	IVMP, PPO, AZA	IMP	No
19	Kondo T et al./2014	58/M	MI, DO, EL, ASAT, Urinary incontinence, scleritis, euphoric		limbic system atrophy resulting in ventricular enlargement	diffuse dominant theta waves with no spike.	33 cells 32% poly Glu normal	GluR N2B, GluRδ2 (CSF, not serum)	NO	1^st^IVMP, PPO 2^nd^INFX	Slight IMO	Yes
23	Zhu Z et al./2018	66/M	Myoclonus, ataxia SA, delirium, Cognitive impairment, MI, Pyramidal Extrapyramidal symptoms	↑	high T2 signals in the bilateral basal ganglia and TL involving the hippocampus 2^nd^atrophy of HC $ white matter lesions	he ‘sphenoid electrodes’ EEG showed high irregular slow waves in the TL	8 cells, Glu normal	No	No	IVMP, PPO, IGs	Slight imp	No
24	Simabukuro MM et al./2016	43/M	FV, A, seizures, MI, ASAT, episcleritis		bilateral TL hyperintensities	N/a	105 cells 77% Glu normal	No	Yes vasculitis	?	No	?
25	Jeon CH/2016	56/M	HA, scleritis, HL, ASAT, dizziness, agitations, hallucinations		N	brief rhythmic spikes and slow wave complexes	25 cells 60% lymph, Glu normal	No	No	IVMP, PPO	No	
28	Ahn S et al./2017	33/M	HA, MI, C, SA	↑	1^st^: FLAIR, T1+Gd high signal with enhancement in cerebral cortex, BG, DWM 2^nd^: both TL, atrophy	No	20 cells, LY pleocytosis Glu N/a	N/a	No	1^st^: IVMP, PPO, AZA2nd: mycophenolate, CY	IMP	Yes
26	Godsave C et al./2018	70/M	FV, ASAT, HA, C, SA, paresthesia	↑	MRI vascular white matter lesions and moderate atrophy	N/a	55 cells Lymph, Glu N/a	No	No	IVMP, PPO, IGs, Mycophenolate mofetil	IMP	Yes
22	Angkodjojo S et al./2020	66/M	C, EL, ASAT, hyperactivity, DO, MI, aggression, Weight loss Episcleritis, confabulation	↑	whole-body PET/CT scan with ^18^F-fluorodeoxy glucose (FDG) was done instead which revealed a focus of intense metabolic activity in the left hippocampal region	Diffuse slowing of background activities to 1–2 Hz	48 cells 74% lymph Glu normal	No	No	IVMP, PPO, CY	IMP	No
	Our patient	(71/M)	Cognitive impairment, MI, EL, DO, personality changes		T2, T2-FLAIR hyperintensities in bilateral medial TL, sub-cortical and DWM and TL atrophy	No pathological findings	LY pleocytosis	No	N/a	IVMP, PPO, CY, AZA	IMP	No

Limbic encephalitis and RP.

M: male; HL: sensorineural hearing loss; FV: fever; HA: headache; ASAT: auricular swelling and tenderness; CJ: conjunctivitis; MI: memory impairment; AG: arthralgia; C: confusion; DO: disorientation; SA: speech abnormalities; EL: emotional liability; FLAIR: Fluid attenuated inversion recovery; HC: Hippocampi; AB: amygdaloidal bodies; PVWM: periventricular white matter; DWM: deep white matter; TL: temporal lobe; IVMP: intravenous methylprednisolone; PPO: prednisone per os; AZA: azathioprine; INFX: infliximab; CY: cyclophosphamide; IGs: immunoglobins; IMP: improvement.

As shown in **Table 1**, the mean age of RP patients with a syndrome of limbic encephalitis is 59 years (range 43–72 years). There is a pronounced male predominance with no female cases of limbic encephalitis due to RP reported in medical literature. This is an interesting finding considering that RP is known to present in similar rates in both genders.

Clinical presentation encompasses heterogenous features, including memory loss, behavioural changes, disorientation, apathy, extrapyramidal symptoms, and speech difficulties. Although seizures are a frequent symptom of limbic encephalitis, they are reported from only three authors. All patients indicated increased inflammatory markers and mononuclear pleocytosis in CSF, with only one reporting polymorphonuclear predominance.

The preferred imaging modality was MRI, whilst in one case report PET/CT was used. MRI studies demonstrated mostly lesions in the temporal lobes, hippocampi, basal ganglia, as well as the deep brain white matter. A common late finding was atrophy of the affected structures. Of note, in two case reports initial MRI scans were free of abnormal findings, highlighting the importance of follow-up imaging. We too found the temporal lobes and hippocampi to be the most affected sites. However, our patient also displayed a progressive confluence of vasculitic lesions located in the periventricular white matter, deep gray matter and subcortical areas mimicking small-vessel disease. Eventually, the progressive atrophy of the medial temporal structures was responsible for the most devastating sequelae in our patient involving memory function.

As far as the CSF analysis is concerned, the available data from the most cases demonstrate pleocytosis with lymphocytic predominance, increased protein levels and normal glucose concentration. Our patient’s marginally low CSF glucose levels from the first lumbar puncture was an unexpected finding. Nevertheless, the negative infectious disease workup along with the second lumbar puncture’s findings (normal CSF glucose concentration) made the possibility of a CNS infection seem more distant.

Additionally, nine out of twelve reported cases tested antibodies for paraneoplastic and/or autoimmune encephalitides. It is important to note that only Kashihara et al. found a patient positive for GluR N2B antibodies, both in CSF and serum, while Kondo et al. identified GluR N2B and GluRδ2 only in CSF (22; 25). These antibodies, which are subunits of the NMDA receptor, have been associated with other forms of limbic encephalitis, such as non-paraneoplastic, non-herpetic and ovarian teratoma associated limbic encephalitides. Although we did not test for these antibodies directly, the lack of positive NMDAR antibodies in our patient strongly advocates against their presence. The role of NMDAR subunits and their relation to central nervous system involvement in RP, remains to be elucidated.

Treatment of RP associated limbic encephalitis is empirical, based on previous reports. The majority of patients are initially treated with high dose corticosteroids showing good response.^18,20,27^ Nevertheless, a small number of cases report slight improvement or failure of initial treatment.^18,22^ Fewer reports exist regarding the treatment of refractory disease. Infliximab, cyclophosphamide, and IVIg have all been tried giving inconsistent results.

Accordingly, we chose glucocorticoids as initial therapy. However, later on, radiological progression of the disease necessitated the use of 2^nd^ line drugs. We chose cyclophosphamide as induction therapy, followed by azathioprine for long-term treatment, both well studied therapies for refractory autoimmune encephalitides and other systemic rheumatological diseases with neuropsychiatric manifestations (eg, systemic lupus erythematosus).^31^ This therapeutic intervention was associated with decreased number of lesions depicted serially on MRI imaging, disappearance of pleocytosis in CSF, as well as significant improvement in neuropsychological testing.

In conclusion, we presented a case of relapsing polychondritis with a late presentation on CNS involvement that traversed the clinical and imaging spectrum of the disease displaying features of limbic encephalitis and small-vessel vasculitis. Importantly, we share our experience of effectively treating refractory limbic encephalitis in the context of RP with the use of cyclophosphamide and azathioprine intending to contribute to the limited data available so far in the literature.
